# Invulnerability of the Urban Agglomeration Integrated Passenger Transport Network under Emergency Events

**DOI:** 10.3390/ijerph20010450

**Published:** 2022-12-27

**Authors:** Peng Wu, Yunfei Li, Chengbing Li

**Affiliations:** 1 School of Traffic and Transportation, Beijing Jiaotong University, Beijing 100044, China; 2 Transportation Institute, Inner Mongolia University, Hohhot 010021, China

**Keywords:** public safety, integrated transportation, complex network theory, passenger transport network, emergency, invulnerability, resilience, urban agglomeration

## Abstract

Urgent natural environmental events, such as floods, power failures, and epidemics, result in disruptions to the traffic system and heavy disturbances in public requirements. In order to strengthen the ability of the transport network to handle urgent natural environmental issues, this paper simulates the disruption situation of traffic stations in the urban agglomeration by attacking nodes, and evaluates the ability of the transport network to resist disruptions (i.e., invulnerability). Firstly, the model of the urban agglomeration integrated passenger transport network is established based on complex network theory. The highway network, railway network, and coupling network are combined into a multi-layer network space structure, and the edge weight is calibrated by travel time and cost. Secondly, the invulnerability simulation process including multiple attack modes under random and deliberate attack strategies is sorted out. By improving the traditional network efficiency indicator, the network impedance efficiency indicator is proposed to measure network performance, and the network relative impedance efficiency indicator is used to evaluate network invulnerability and identify key nodes. Finally, Chengdu–Chongqing urban agglomeration is taken as a case study. The results show that the network does not collapse quickly and it shows certain invulnerability and robustness under continuous random attacks. Network performance and invulnerability are not necessarily positively correlated. The failure of individual nodes that are small in scale but act as transit hubs may significantly degrade the network performance. The identified key nodes have significance for guiding the construction, maintenance, and optimization of the urban agglomeration passenger transport network, which is conducive to promoting public safety.

## 1. Introduction

Traffic environment flexibility or vulnerability are closely related to public safety. A harmonious traffic environment free from natural disasters and infectious diseases can ensure the safe and efficient operation of the transportation network. However, emergency events such as natural disasters, harsh environments, and infectious diseases have always been potential threats to the transportation network, which may result in the destruction or closure of traffic stations, thereby reducing transport efficiency. Studying the phenomenon of traffic operation disruptions under the impact of urgent natural environmental events is conducive to identifying potential weaknesses and promoting public safety. Therefore, it is necessary to study the ability of the transport network to resist disruptions (i.e., invulnerability).

How to simulate disruptions in the traffic system is a problem worth exploring. Based on complex network theory, traffic station disruptions under emergency events can be simulated by attacking nodes in the network. The more impact a failed node has on network efficiency, the more critical it is. Through this method, stations that have a high impact on the efficiency of the transport network after failure will be identified, which can provide suggestions for the maintenance and optimization of the traffic system and help enhance coordination between the environment and transportation.

Urban agglomeration is the focus of the economy and population in many countries, and also the trend of urbanization development in the world. Intercity communication is becoming more and more frequent, and the urban agglomeration passenger transport network is becoming larger and larger. As the urban agglomeration spans multiple administrative regions, the planning and development of the passenger transport network are not close, so there are some weak links. In order to strengthen the ability of the transport network to cope with emergency events, it is necessary to evaluate the invulnerability of the highway–railway integrated passenger transport network in the urban agglomeration.

Invulnerability refers to the ability to resist disruptions. Albert et al. [1] conducted research on network invulnerability, and found that some complex networks show surprising robustness to random attacks and are very vulnerable to deliberate attacks. The urban agglomeration transportation network shows strong robustness to random attacks and exhibits vulnerability to deliberate attacks [2]. For random attacks and deliberate attacks, the former uses network robustness analysis and the latter uses network vulnerability analysis [3]. It can be considered that network invulnerability is the sum of robustness to random attacks and vulnerability to deliberate attacks. Therefore, this paper adopts random and deliberate attack strategies to evaluate network invulnerability.

Up until now, there has been extensive research on transportation networks based on complex network theory. Xu et al. [4] evaluated the connectivity of China’s high-speed railway system. Luo et al. [5] conducted a case study on the tram networks of the Hague and Amsterdam. Wang et al. [6] estimated a statistical model to identify the relationship between transfer flow and the network properties in a joint bus and metro network. Zhang et al. [7] applied complex network theory to analyze the evolution of the risk propagation airport network. Zhou et al. [8] made use of complex network science indicators with spatial cognition weighted accessibility combining full urban rail network, road network, and pedestrian network. Yang et al. [9] constructed the Maritime Silk Road shipping network based on complex network theory. Wu et al. [10] attempted to use complex network theory and spatial autocorrelation analysis approaches to examine structural properties of bike-sharing systems. Abdelaty et al. [11] adopted complex network theory in the context of the bus transit network. Bombelli et al. [12] presented a complex network analysis of the air transport network using air cargo. Soh et al. [13] contributed a complex weighted network analysis on the travel routes of the Singapore rail and bus transportation systems. Xing et al. [14] analyzed both the topological and dynamic properties of the Shanghai rail transit system network by using complex network theory. Jia et al. [15] proposed a new method considering station hybrid influence and passenger flow to identify key stations in the whole bus network, based on complex network theory. Wu et al. [16] proposed a key flight conflict node identification method based on complex network theory and the Analytic Hierarchy Process (AHP) entropy weight method. Liu et al. [17] viewed the integration process of air traffic into a temporally connected network as a percolation process, and measured the global reliability of air traffic. Zhang et al. [18] set up a complex network in China railway express transport and identified important logistics nodes. Hamedmoghadame et al. [19] introduced a percolation-based network analysis framework and theoretically identified bottleneck links, and then the effectiveness of the framework was demonstrated based on large-scale real transportation networks. It can be seen from the existing research that the application of complex network theory in the modeling and network analysis of real complex traffic systems is a research hotspot in the field of transportation, and the application of complex network theory in transportation networks is becoming more mature and appropriate.

Based on complex network theory, many studies have been carried out to investigate the invulnerability of transportation networks. Angeloudis et al. [20] analyzed the robustness of the subway network from the perspective of network topology connectivity. Liu et al. [21] analyzed the cascading failure resistance of the urban rail traffic network under different failure strategies. Li et al. [22] proposed a method to analyze the vulnerability of a public transport system composed of two or more transport subsystems and identified the critical area within the system based on the data of the running timetable and position coordinates. Yap et al. [23] developed a methodology to identify the most vulnerable links in the total, multi-level public transport network. Sohn [24] conducted an analysis to assess the significance of highway network links in Maryland under flood damage. Zhang et al. [25] analyzed the networked characteristics of three metro networks, and two malicious attacks were employed to investigate the vulnerability of metro networks based on connectivity vulnerability and functionality vulnerability. Latora et al. [26] discussed a general method to find the critical components of an infrastructure network and analyzed the vulnerability and protection of infrastructure networks. Cats et al. [27] adopted a complex network theory approach, investigating network performances under alternative sequential disruption scenarios. The research on transport system vulnerability is now a mature field with a developed methodology and a large amount of research findings with large potential practical usefulness [28]. Mou et al. [29] assessed the resilience of the maritime crude oil transportation network from both qualitative and quantitative perspectives with the help of complex network metrics and a resilience model. Xu et al. [30] conducted an empirical study on the resilience of five metro or subway systems selected globally. Hassan et al. [31] quantified the robustness of the City of Minneapolis bus transit network. Sun et al. [32] simulated the vulnerability of the Beijing urban rail transit network under malicious attacks and random attacks. Accurately identifying the key nodes of the road network and focusing on its management and control is an important means to improve the robustness and invulnerability of the road network [33].

Studies on the invulnerability of transportation networks based on complex network theory mainly focus on urban buses, rail transit, and high-speed rail and aviation networks, while there are few studies on the invulnerability of highway and railway passenger networks in the urban agglomeration. Some scholars have paid attention to the invulnerability of the integrated passenger transport network in the urban agglomeration [2]. Liu et al. [34] examined the cascading failure in both the syncretic and single railway networks in the Chengdu–Chongqing urban agglomeration. Chen et al. [35] developed vulnerability and resilience assessment models to investigate the influences on the integrated transportation network model of Guangdong–Hong Kong–Macao Greater Bay Area (GBA). Liu et al. [36] analyzed the vulnerability of the planning rail transit network in the Beijing–Tianjin–Hebei urban agglomeration. Li et al. [37,38] built the model of urban agglomeration compound traffic network, and studied the cascading failure invulnerability of the urban agglomeration composite traffic network. However, this method only combines the road and railway stations that are close to each other and ignores the influence of urban traffic transfer. Guan et al. [39] discussed the key issues and prospects in transportation system engineering in detail, and believed that the complex characteristics of the multi-layer integrated transportation network structure and its dynamic process need to be studied in depth.

To sum up, the existing studies of transport network invulnerability focus more on network topological properties and passenger flow factors, and lack research on network travel impedance (i.e., network functional properties). Moreover, there are few studies on the invulnerability of the urban agglomeration passenger transport network, and the multi-layer spatial structure of the urban agglomeration highway-railway integrated passenger transport network is not clear yet. Therefore, the multi-layer spatial structure of the highway–railway integrated passenger transport network in the urban agglomeration is illustrated from the perspective of coupling. On this basis, random and deliberate attack strategies are used to study the dynamic process of network travel impedance variation, and then the impedance invulnerability of the network is evaluated.

The contents of this paper are as follows: The modeling method and the invulnerability analysis method of the urban agglomeration integrated passenger transport network are proposed in Section 2. The integrated passenger transport network in Chengdu–Chongqing urban agglomeration is taken as a case study in Section 3. The conclusions of this paper and the prospect of further research are summarized in Section 4.

## 2. Methods

### 2.1. The Modeling Method

At present, the application of complex network theory in the urban agglomeration integrated passenger transport network has some deviations from the actual network. First of all, the influence of urban traffic transfer is ignored. Some intercity traffic stations can be connected through urban traffic, walking, and other ways to generate coupled networks. In addition, more consideration is given to the topology characteristics of the network itself. Indicators such as degree, mediations, average path length, network efficiency, and connected subgraphs ignore the functional properties of the network. Therefore, it is necessary to build a more realistic urban agglomeration integrated passenger network model. The general steps are as follows. Step 1: the highway network and the railway network are combined to construct the intercity network. Step 2: the intercity network and the coupling network are combined to construct the integrated network. Step 3: the network functional attribute is calibrated, that is, the travel impedance. The specific methods are as follows.

#### 2.1.1. The Intercity Network

**Figure 1:**
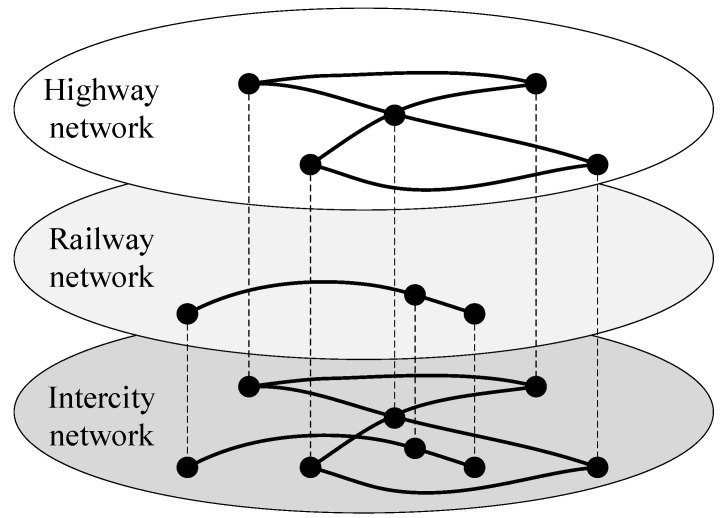
Schematic diagram of the intercity network.

Based on complex network theory, coach stations are abstracted as nodes in the network. Due to the complexity of highway passenger transport, it is extremely difficult to obtain specific driving routes. Therefore, if a single bus ticket can be purchased between two stations, it is considered that there is a connecting edge between the two stations, so as to construct the highway passenger transport network (highway network). On the basis of complex network theory and the Space L [40] method, railway passenger stations are abstracted as nodes in the network, and railway actual routes are abstracted as connecting edges in the network, so as to construct a railway passenger transport network (railway network). The highway passenger transport network and the railway passenger transport network are combined to construct an intercity passenger transport network (intercity network). The schematic diagram of the intercity network is shown in Figure 1.

Nodes in the intercity network are called intercity stations, and edges are called intercity edges. It is worth noting that although the highway network and the railway network form the intercity network, they cannot communicate with each other and are still independent. This is because the exchange of passenger flow between the bus station and the railway station mainly relies on urban transportation, walking, private cars, and so on. Therefore, it is necessary to consider the transfer within the city to connect the highway network with the railway network, so as to jointly establish an integrated passenger transport network in the urban agglomeration.

#### 2.1.2. The Integrated Network

**Figure 2:**
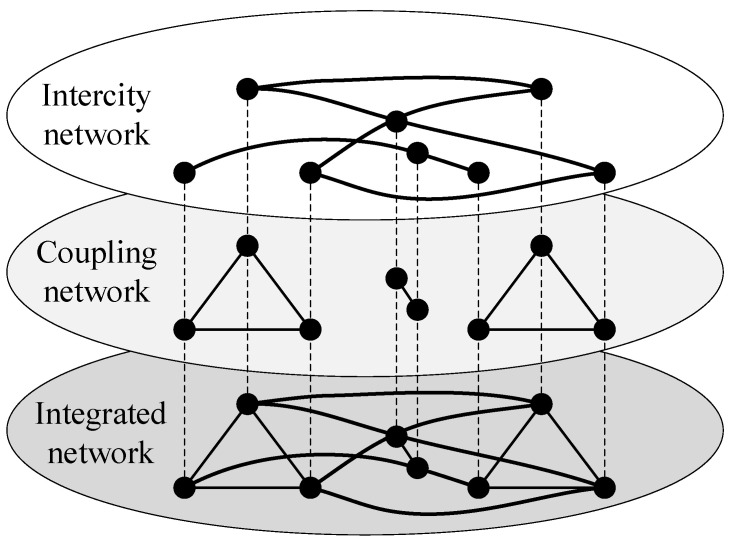
Schematic diagram of the integrated network.

Coupling edges are added between intercity stations with convenient transfers such as urban public transport, walking, and private cars to build a coupling network. The intercity network and the coupling network are combined and superimposed into an integrated network, which is the spatial structure of the urban agglomeration integrated passenger transport network. The schematic diagram of the integrated network is shown in Figure 2.

According to the spatial structure of the integrated network shown in Figure 1 and Figure 2, the model of the urban agglomeration integrated passenger transport network considering travel impedance is denoted by G={V,B,O,W,H}, where V means the set of network nodes, V={1,2,3,⋯,N} and N indicates the total number of nodes. $B={\left(b_{ij}\right)}_{N\times N}$ represents the adjacency matrix of the intercity network. If there is an intercity edge between nodes, then $b_{ij}\wedge b_{ji}=1$. If there is no intercity edge, then $b_{ij}\wedge b_{ji}=0$. $O={\left(o_{ij}\right)}_{N\times N}$ represents the adjacency matrix of the coupling network. If there is a coupling edge between nodes, then $o_{ij}\wedge o_{ji}=1$. If there is no coupling edge, then $o_{ij}\wedge o_{ji}=0$. $W={\left(w_{ij}\right)}_{N\times N}$ denotes the edge weight matrix, which is calibrated by the travel impedance. $H=\{h_{1},h_{2},h_{3},\cdots ,h_{N}\}$ denotes the set of node weights, and the node weight is calibrated by the sum of all edge weights of the node.

#### 2.1.3. Network Functional Properties

The edge weight matrix W can represent the virtual direct structure of the network. Therefore, some virtual direct edges with calibrated edge weights appear accordingly. The existence of virtual direct edges depends on whether a single ticket can be queried and purchased between two stations. First, we obtain the most frequent fare and time of direct single tickets between two stations, and construct the travel impedance function $f(w_{ij})$, as shown in the following equation:

(1) $$f(w_{ij})=c_{ij}+v_{ij}t_{ij}$$

where $c_{ij}$ represents the fare between node i and node j, $t_{ij}$ denotes the time between node i and node j, and $v_{ij}$ means the time value mean between node i and node j. In addition, if there is no single ticket, the travel impedance is 0.

The adjacency matrix of the integrated network $BO={\left(bo_{ij}\right)}_{N\times N}$ are superposed and merged by the adjacency matrix of the intercity network B and the adjacency matrix of the coupling network O, which can represent the weightless topology structure of the integrated passenger transport network in the urban agglomeration (the weightless topology network). The weighted adjacency matrix of the integrated network $BOW={\left(bow_{ij}\right)}_{N\times N}$ is obtained by the dot product of the adjacency matrix of the integrated network BO and the edge weight matrix W, which can denote the weighted topology structure of the integrated passenger transport network in the urban agglomeration (the weighted topology network).

### 2.2. The Invulnerability Analysis Method

#### 2.2.1. Attack Modes

Random and deliberate attack strategies are selected. The random attack strategy only includes the random attack. The deliberate attack strategy includes the node-numbered attack, the node-weighted attack, and the node-single attack. The random attack refers to a cumulative attack mode that randomly determines the attack sequence of nodes. The node-numbered attack is a cumulative attack mode that is sorted by node number from small to large. The node-weighted attack refers to a cumulative attack mode that is sorted by node weight, from large to small. The node-single attack is a single-node attack based on the node number, from small to large. After each node is attacked, the network is restored to the initial state before the next node is attacked.

After a node is attacked and fails, the node and the outgoing and incoming edges connected to the node are deleted in the weighted topology network. For example, after a node fails, in the weighted adjacency matrix of the integrated network $BOW={\left(bow_{ij}\right)}_{N\times N}$, for any j∈V, $bow_{ij}\wedge bow_{ji}=0$. The weighted adjacency matrix of the integrated network is constantly updated as nodes fail. Assuming that the failure process of each node is an iterative process, $BOW^{k}$ represents the weighted adjacency matrix after the k iteration, and $BOW^{0}$ represents the initial weighted adjacency matrix.

#### 2.2.2. Invulnerability Evaluation Indicators

In complex network theory, the network efficiency is a common indicator to measure network performance, which is shown in the following equation:

(2) $$E=\frac{1}{N(N-1)}\sum_{i\neq j}\frac{1}{d_{ij}}$$

where E represents the network efficiency, N denotes the number of nodes, and $d_{ij}$ represents the number of edges on the shortest path between node i and node j.

The traditional network efficiency indicator reflects the topology of the network without considering the functional properties of the network. Therefore, some scholars have started to construct weighted network efficiency indicators. For example, the authors of [41] proposed a novel indicator of metro network service efficiency considering the influence of line flows, which is used to measure the network performance. Therefore, the travel impedance considered in this paper can also be used as a weighted factor to improve the traditional network efficiency indicator. Firstly, the impedance efficiency $I_{ij}$ between nodes i,j is defined, which is shown in the following equation:

(3) $$I_{ij}(k)=1/\sum_{(i^{\prime },j^{\prime })\in L_{ij}^{k}}bow_{i^{\prime }j^{\prime }}^{k}$$

where $I_{ij}(k)$ represents the impedance efficiency between node i and node j after the k iteration, and $L_{ij}^{k}$ denotes the set of edges of the shortest path between node i and node j after the k iteration. For example, the shortest path between node $v_{1}$ and node $v_{6}$ is $v_{1}\to v_{3}\to v_{6}$, then $L_{1,6}^{k}=\{(1,3),(3,6)\}$. If there is no shortest path, then $I_{ij}(k)=0$. $bow_{i^{\prime }j^{\prime }}^{k}$ represents the edge weight between node $i^{\prime }$ and node $j^{\prime }$ after the k iteration.

Network impedance efficiency I is defined, which is shown in the following equation:

(4) $$\begin{array}{l} I(k)=\frac{1}{N(N-1)}\sum_{i\neq j}I_{ij}(k)\\ \;=\frac{1}{N(N-1)}\sum_{i\neq j}\left(1/\sum_{(i^{\prime },j^{\prime })\in L_{ij}^{k}}bow_{i^{\prime }j^{\prime }}^{k}\right) \end{array}$$

where I(k) represents the network impedance efficiency after the k iteration.

In order to reflect the performance changes of the network after being disturbed and measure the invulnerability of the network, the invulnerability evaluation indicator of the network’s relative impedance efficiency is proposed, which is shown in the following equation:

(5) $$I^{\prime }(k)=\frac{I(k)}{I(0)}$$

where $I^{\prime }(k)$ represents the relative impedance efficiency of the network after the k iteration and I(0) denotes the initial impedance efficiency of the network.

#### 2.2.3. Invulnerability Simulation Process of Attacking Nodes

The specific steps of the invulnerability simulation process of attacking nodes using MATLAB are as follows:

- Step 1: The network is initialized, after that the initial weighted adjacency matrix $BOW^{0}$ is obtained, and the initial network impedance efficiency I(0) is calculated.
- Step 2: The attack strategies, attack modes, and attack sequence are determined. If it is the random attack strategy, the node number sequence is randomly determined, and the set of the node attack sequence is R=randperm(N). If it is the node-numbered attack in the deliberate attack strategy, the set of the node attack sequence is R=V. If it is the node-weighted attack in the deliberate attack strategy, $\left[H^{\prime },R]=\mathrm{sort}(H,‘\mathrm{descend}’\right)$, the set of the node attack sequence is R.
- Step 3: The set of the attack sequence R is inputted to determine the total number of iterations $k_{\max}=\mathrm{size}(R)$.
- Step 4: k=1, which means attacking node i=R(1).
- Step 5: Node i=R(k) is attacked. The weighted adjacency matrix $BOW^{k}=BOW^{k-1}$, for any j∈V, $bow_{ij}^{k}\wedge bow_{ji}^{k}=0$.
- Step 6: The relative impedance efficiency of the network $I^{\prime }(k)$ is calculated and added to the set $I^{\prime }$.
- Step 7: The judgment of attack end. If $k<k_{\max}$, k=k+1 and go to Step 5. On the other hand, go to Step 8.
- Step 8: The set $I^{\prime }$ is outputted, and the trend chart of the network’s relative impedance efficiency under different attack modes is drawn, and the invulnerability simulation is finished.

**Figure 3:**
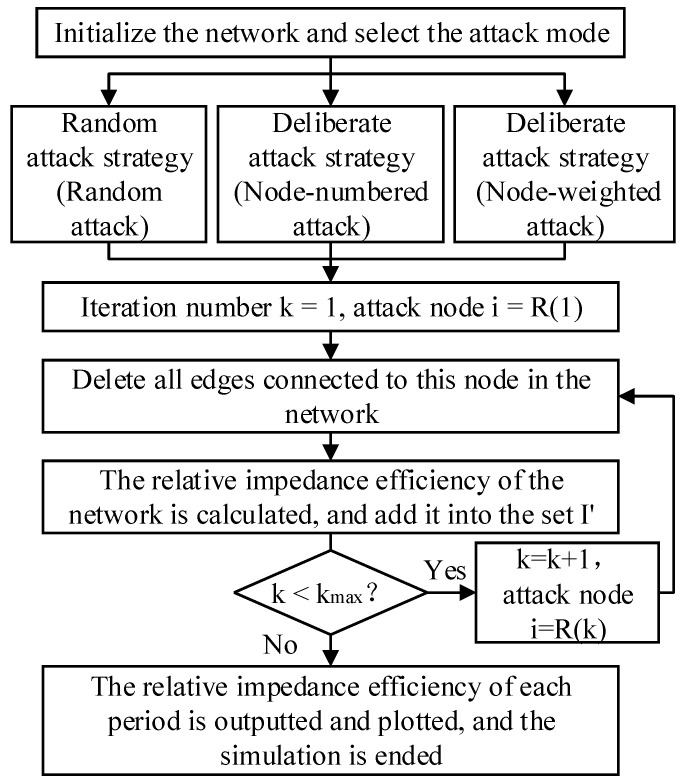
The invulnerability simulation process of attacking nodes.

To sum up, the invulnerability simulation process of attacking nodes is shown in Figure 3.

In addition to the node-numbered attack and node-weighted attack, there is also the node-single attack in the deliberate attack strategy. The node-single attack is attacked one by one according to the node number. However, after each iteration, the network returns to the initial state before the next iteration. The node-single attack can measure the impact on network performance after a node fails.

## 3. The Case Study

### 3.1. The Integrated Passenger Transport Network in Chengdu-Chongqing Urban Agglomeration

According to the “Approval of the State Council on the Development Plan of Chengdu–Chongqing Urban Agglomeration” and the “Outline of the Construction Plan of Chengdu–Chongqing Economic Circle”, it is determined that the Chengdu–Chongqing urban agglomeration includes 16 cities in Sichuan Province, which are Chengdu, Zigong, Luzhou, Deyang, Mianyang, Suining, Neijiang, Leshan, Nanchong, Meishan, Yibin, Guangan, Dazhou, Yaan, and Ziyang, as well as Chongqing’s central city, including new districts for urban development (a total of 12 districts), suburban districts or counties (a total of 27 districts or counties, including Wanzhou, Qianjiang, Liangping, Fengdu, Dianjiang, Zhongxian, etc.), parts of Kaizhou, and parts of Yunyang.

Because of the complexity of road transportation, multiple bus stations in the same city were abstracted into one intercity bus station. So, there were 36 bus stations. The Chongqing highway passenger ticketing website and the Sichuan bus passenger ticketing website were checked node-to-node to know whether the intercity passenger line was open. If so, there was an edge between the two nodes. Based on this method, the adjacency matrix of the Chengdu–Chongqing highway network was constructed, and the Chengdu–Chongqing highway fare matrix was constructed by recording the ticket price with the highest frequency. A total of 73 railway passenger stations in county-level cities were selected. According to the actual route of the railway, the adjacency matrix of the Chengdu–Chongqing railway network was constructed. The intercity stations were numbered consecutively according to highway and then railway to get the node set V={1,2,3,⋯,109}. The adjacency matrix of the Chengdu–Chongqing highway network and railway network were merged into the intercity network adjacency matrix $B={\left(b_{ij}\right)}_{109\times 109}$. The longitude and latitude coordinates of each node were obtained and recorded in the Baidu map picking coordinate system.

The ticket was checked node-to-node on the official website of China Railway 12306. If it existed, a virtual direct edge was added between the two stations, and the fares with the highest frequency and the corresponding times were recorded. If the frequencies were the same, the lowest fare and the corresponding time were recorded. Based on this, the Chengdu–Chongqing railway fare matrix was constructed. As the actual running time of road passenger transportation is extremely difficult to know, in order to correspond to railway passenger transportation, time was not considered for the time being, and the impedance function only considered the fare. The Chengdu–Chongqing highway fare matrix and the Chengdu–Chongqing railway fare matrix were combined into the edge weight matrix $W={\left(w_{ij}\right)}_{109\times 109}$. The weight of each node was the sum of the edge weights of all connected edges of the node, so as to construct the node weight set $H=\{h_{1},h_{2},h_{3},\cdots ,h_{109}\}$.

**Figure 4:**
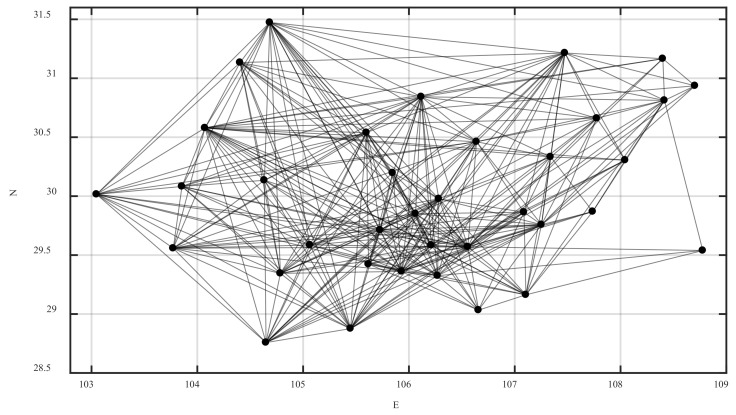
The highway passenger transport network of the Chengdu–Chongqing urban agglomeration.

**Figure 5:**
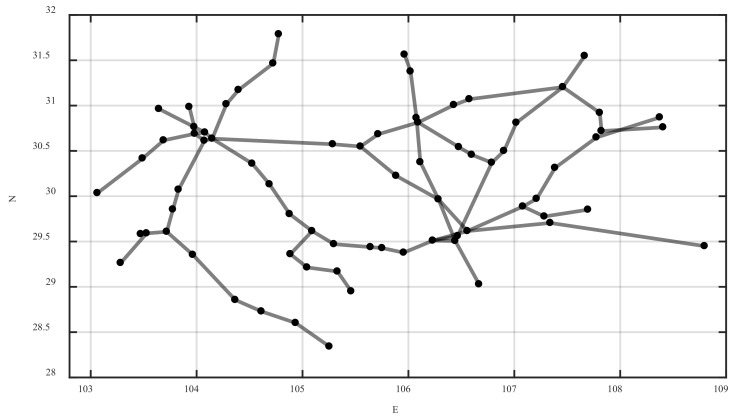
The railway passenger transport network of the Chengdu–Chongqing urban agglomeration.

**Figure 6:**
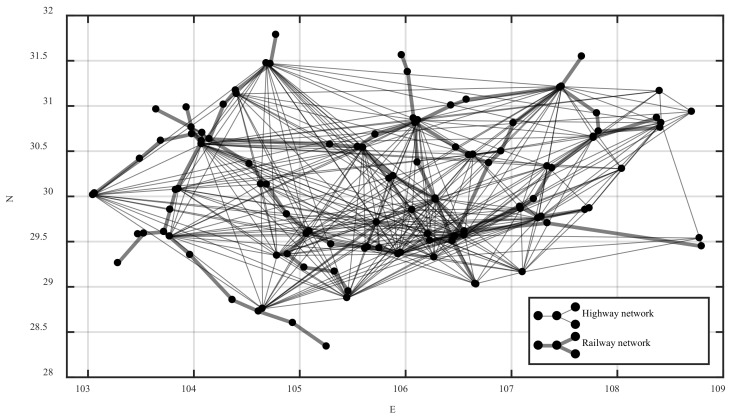
The integrated passenger transport network of the Chengdu–Chongqing urban agglomeration.

The coupling edge adjacency matrix was constructed by adding the coupling edges between intercity stations that were connected by urban public transit, walking, or private cars in the same city. Based on this, the Chengdu–Chongqing urban agglomeration integrated passenger transport network model was constructed. The intercity network adjacency matrix and the coupling network adjacency matrix were combined and superimposed into the integrated network adjacency matrix. The weighted adjacency matrix was obtained by the dot product of the integrated network adjacency matrix and the edge weight matrix. According to the weighted adjacency matrix and the latitude and longitude coordinates of the nodes, the highway, the railway, and the integrated passenger transport network of the Chengdu–Chongqing urban agglomeration was drawn in MATLAB, as shown in Figure 4, Figure 5 and Figure 6, respectively.

In Figure 6, the highway nodes are numbered from 1 to 36, and the railway nodes are numbered from 37 to 109.

### 3.2. Invulnerability Simulation of Attacking Nodes Randomly

**Figure 7:**
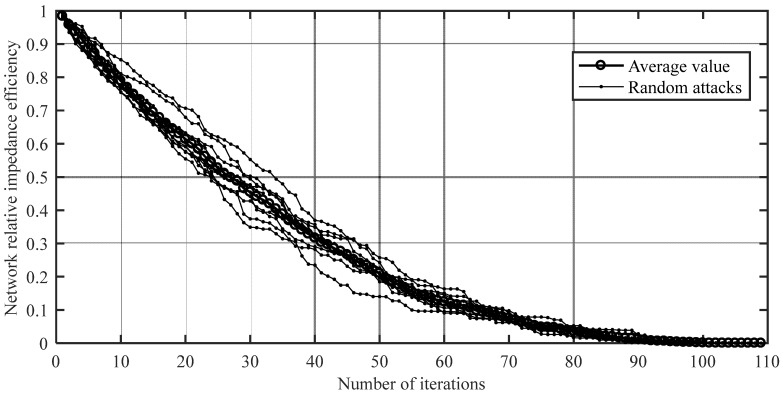
The trend of network performance of attacking nodes randomly.

According to the random attack strategy in the invulnerability simulation process of attacking nodes in Section 2.2.3, 10 random attacks were carried out on the integrated passenger transport network of the Chengdu–Chongqing urban agglomeration, and the average value of 10 random attacks was calculated. The trend of network performance for the attack nodes randomly was obtained, as shown in Figure 7.

As can be seen from Figure 7, in the face of random attacks, the variation of network performance had a similar trend but fluctuated, and the decline rates were all characterized as fast first and then slow. When the attack reached the first, second, third, fourth, and fifth nodes (the number of iterations is k=1,2,3,4,5), the network performance dropped by 1.63%, 4.16%, 6.26%, 8.63%, and 11.23%, respectively, on average. When the attack reached the 10th node (the number of iterations is k=10), the network performance dropped by 14~25%, with an average drop of 21.09%; when the attack reached the 20th node (the number of iterations is k=20), the network performance dropped by 29~45%, with an average drop of 38.61%; when the attack reached the 40th node (the number of iterations is k=40), the network performance dropped by 63~77%, with an average drop of 68.33%. Although random attacks had strong uncertainties, the results of multiple random attacks show that the network did not collapse rapidly in the face of continuous random attacks, and showed a certain ability to resist destruction and robustness, but the network performance degraded significantly.

### 3.3. Invulnerability Simulation of Attacking Nodes Deliberately

**Figure 8:**
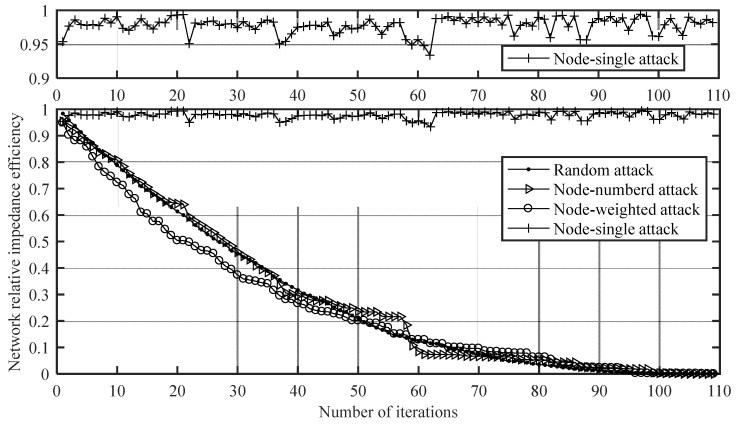
The trend of network performance of attacking nodes deliberately.

According to the deliberate attack strategy in the invulnerability simulation process of attacking nodes in Section 2.2.3, the cumulative attack according to the numbered sequence (node-numbered attack) and the cumulative attack in descending order of node weight (node-weighted attacks), and the node-single attack were carried out on the network. Taking the average value of 10 random attacks as a reference (random attack), the trend of network performance of attacking nodes deliberately is shown in Figure 8.

As can be seen from Figure 8, the network impedance efficiency dropped sharply in the early stage of the node-weighted attack. So, the failure of nodes with high weights may have a great impact on network performance. In the first 50 iterations of the node-weighted attack, the network performance was obviously lower than that of the random attack. In the last 59 iterations, the network performance was slightly higher than that of the random attack. It can be seen that even if the performance of the network was lower, its invulnerability may be better than a network with a better performance. In the node-numbered attack, the network impedance efficiency dropped sharply at the 22nd, 37th, 38th, 58th, and 59th iterations. The attacked nodes were Chengdu Highway Station, Chongqing North Railway Station, Chongqing West Railway Station, Chengdu Railway Station, and Chengdu East Railway Station. Therefore, the failure of a large-scale site may have a great impact on network performance.

**Figure 9:**
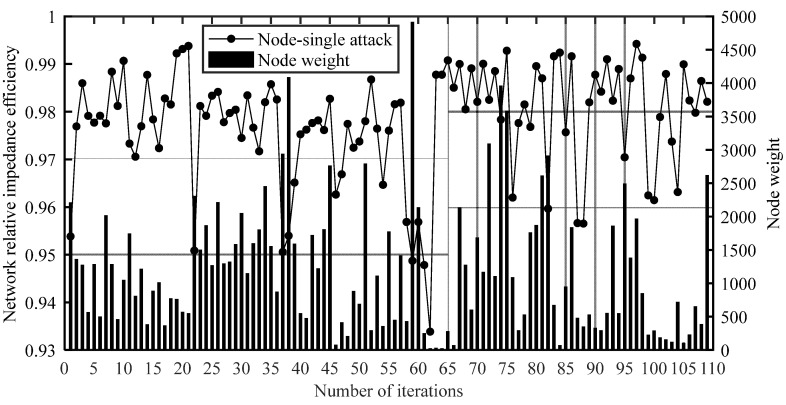
Comparison of node weight and node-single attack results.

In order to explore whether the failure of a node with a higher weight will have a greater impact on network performance, the weight of each node is compared with the result of the node-single attack, as shown in Figure 9.

As can be seen from Figure 9, the failure of some nodes with high weights will have a great impact on network performance, such as nodes 38 and 59, which were Chongqing West Railway Station and Chengdu East Railway Station, respectively. However, there was no significant relationship between the node weight and the result of the node-single attack. Individual nodes had higher weights but less impact on network performance, while individual nodes had lower weights but a greater impact on network performance. For example, the weight of node 75 was high, but the network performance dropped less after the node failed; the weight of node 61 was very low, but the network performance dropped more after the node failed.

In the node-single attack, obviously the failure of some nodes will greatly affect the network impedance efficiency. Therefore, the seven nodes that greatly affected the network performance after the node-single attack are shown in Table 1.

As can be seen from Table 1, Xipu Railway Station had the greatest impact, and it is an important starting station, intermediate station, and transfer hub of the Chengdu Metropolitan Railway Chengguan Line and Pengzhou Branch Line. Therefore, the failure of Xipu Railway Station will cause itself, Dujiangyan Railway Station, Pengzhou Railway Station, etc., to be unable to be reached, and then the impedance efficiency between a large number of node pairs will become zero, and the network impedance efficiency will drop sharply. It can be seen that the failure of some important transfer hubs will have a great impact on network invulnerability. Moreover, the stations in Table 1 are all stations in the central urban areas of Chengdu and Chongqing. Most of them have high node weights and are recognized as large-scale stations, reflecting the dual core status of Chengdu and Chongqing in the Chengdu–Chongqing urban agglomeration.

In addition, the seven nodes that have less impact on the network performance after the node-single attack are shown in Table 2.

Combined with the integrated passenger transport network of Chengdu–Chongqing urban agglomeration in Figure 4, it can be seen that most of these stations with less impact on the network performance in Table 2 were located at the edge of the network and had few edges, which conforms to the actual situation and reflects the correctness of simulation to some extent.

## 4. Conclusions

This paper innovatively proposes the modeling method and the invulnerability analysis method of the urban agglomeration integrated passenger transport network based on complex network theory. The method of attacking nodes is used to simulate the disruption situation of traffic stations under urgent natural environmental events. The invulnerability simulation including multiple attack modes under random and deliberate attack strategies is carried out in a case study. The conclusions are as follows:

(1) Under multiple random attacks, the network performance changes in a similar trend. The network does not collapse quickly, showing certain damage resistance and robustness, but the network performance will be greatly affected.
(2) The network performance in the early stage of the node-weighted attack is lower than that of the random attack, and the network performance in the later stage is slightly higher than that of the random attack. Therefore, a network with a lower performance may be better than a network with a higher performance in terms of invulnerability, and the network performance and invulnerability are not necessarily positively correlated.
(3) There is no obvious relationship between the node weight and the node-single attack result. The failure of some large-scale nodes may have a greater impact on the network performance, and the failure of individual nodes on small scales may also have a greater impact on the network performance, which reflects the complexity of the integrated passenger transport network in the urban agglomeration, so it is necessary to strengthen the supervision and maintenance of these stations or coordinate with other modes of transport for optimization.
(4) The nodes in Chengdu and Chongqing play an important role in maintaining the network performance, which reflects the dual core status of Chengdu and Chongqing in the Chengdu–Chongqing urban agglomeration. It also shows from the side that the integrated development of the Chengdu–Chongqing urban agglomeration is not balanced, and there is a collapse in the middle, so it is necessary to strengthen the development and support of other cities.

Through these methods, key traffic stations (i.e., vulnerable nodes potentially affecting public safety) in the urban agglomeration will be identified, which can inform the transportation planning department and help transportation planners in tactical planning decisions. Improving the ability of these key traffic stations to respond to urgent natural environmental events (i.e., ensuring their normal operation under emergency events) is conducive to maintaining the transport efficiency of the urban agglomeration traffic system and promoting public safety. In addition, urgent natural environmental events would disrupt not only traffic stations, but also traffic routes. Therefore, further research could simulate the situation of edge failure based on the methods of this paper. Moreover, the disturbances on passenger demand under traffic disruptions are also worth studying in the future, including passenger travel path replanning and passenger flow diffusion.

## Figures and Tables

**Table 1:** Nodes that have a significant impact on network performance.

Sort (Number)	Node	Relative Impedance Efficiency
1 (62)	Xipu Railway Station	0.933869143
2 (61)	Chengdu West Railway Station	0.947845321
3 (59)	Chengdu East Railway Station	0.948733038
4 (37)	Chongqing North Railway Station	0.950581884
5 (22)	Chengdu Highway Station	0.950873039
6 (01)	Chongqing Highway Station	0.953840324
7 (38)	Chongqing West Railway Station	0.953985546

**Table 2:** Nodes that have a small impact on network performance.

**Sort (Number)**	**Node**	**Relative Impedance Efficiency**
109 (97)	Xingwen Railway Station	0.994211444
108 (21)	Yunyang Highway Station	0.993794890
107 (20)	Kaizhou Highway Station	0.993136849
106 (75)	Jiangyou Railway Station	0.992790545
105 (84)	Ebian Railway Station	0.992396994
104 (19)	Zhongxian Highway Station	0.992233333
103 (83)	Qianwei Railway Station	0.991630945

## Data Availability

The data that support the findings of this study are available from the corresponding author upon request.
